# BACE1: from biomarker to Alzheimer’s disease therapeutical target

**DOI:** 10.18632/aging.203064

**Published:** 2021-05-11

**Authors:** Carlo Cervellati, Giuseppe Valacchi, Giovanni Zuliani

**Affiliations:** 1 Department of Translational Medicine and for Romagna, University of Ferrara, Ferrara, 44121, Italy; 2 Department of Neuroscience and Rehabilitation, University of Ferrara, Ferrara, 44121, Italy; 3 Plants for Human Health Institute, Animal Science Department, NC State University, Kannapolis, NC, 28081, USA; 4 Department of Food and Nutrition, Kyung Hee University, Seoul, South Korea

**Keywords:** Alzheimer’s disease, amyloid- β, BACE1, blood-based biomarkers

Amyloid precursor protein (APP) cleaving enzyme-1 (BACE1) is the rate-limiting enzyme for amyloid-β peptides (Aβ) generation in the brain [1]. The BACE1 cleavage of APP results in a deviation of the physiological non-amyloidogenic pathway, driven by α-secretase, leading to the formation of two peptides, the soluble ectodomain APPsβ and the membrane−bound C-terminus (C99). The latter is further processed by γ-secretase to generate Aβ40 and Aβ42. Aβ42 is highly neurotoxic and aggregates to form the “senile plaques”, the neuropathological hallmarks of Alzheimer's disease (AD). As postulated by the “amyloid cascade hypothesis”, the accumulation of Aβ42 is a downstream event in AD; it precedes and most likely contributes to triggering hyperphosphorylation of tau, thus producing the intracellular neurofibrillary tangles (NFT) [1]. This second AD core pathology, together with Aβ accumulation, contributes to the cascade of aberrant events, leading to brain synaptic and neuronal loss [1].

BACE1 is central in the metabolism of Aβ; therefore, the inhibition of its expression/activity results in a decrease of Aβ40/Aβ42 production. BACE1 concentration and activity have been found to be elevated brain and cerebrospinal fluid (CSF) of AD patients compared with normal individuals. Intriguingly, two recent large population studies [2,3] from our group demonstrated that this alteration is also reflected in periphery, with a 30% increase in serum BACE1 activity in AD patients compared to controls. This finding is supported by some elegant investigations from Hampel’s group [4,5], showing both a correlation of peripheral BACE1 with well-established AD markers (CSF Aβ and t-Tau), and with axonal degeneration and brain atrophy in individuals at high risk for AD.

Despite this body of evidence, defining plasma/serum BACE1 activity as a strong candidate biomarker for the diagnosis of AD might be still premature. Anyway, compared to available CSF and imaging biomarkers, it would be much more suitable for routine clinical practice (lower invasiveness, high cost effective) [6]. However, the diagnostic accuracy we have detected is acceptable (77%; sensitivity/specificity: 73/70%), but still low compared with other blood-based biomarkers such Aβ42 (or Aβ 42/40 ratio), phosphorylated tau181, and total tau [6]. This doesn’t preclude the possibility of using BACE1 as biomarker for large-scale screening in primary care settings; indeed, its unexpensive assay could provide information about one of the most prominent abnormalities driving AD development. In our opinion, combining BACE1 serum measurement with other markers reflecting different coexistent pathological AD features (i.e. inflammation, vascular dysregulation, neurodegeneration) could be useful in multiple clinical contexts, including trial enrollment and monitoring.

AD remains a drug-orphan disease, and BACE1 is one of the pharmacological target in trials for this disorder [7]. Unfortunately, the majority of these trials highlighted a high failure rate for several drug candidates, including BACE1 inhibitors [7]. The failure of these and other anti-amyloid therapies has represented the most critical challenge to the “amyloid cascade hypothesis”. Indeed, while for other well-known enzymes inhibitors (e.g. statins for HMG-CoA reductase to reduce LDL-cholesterol and cardiovascular disease) the “theory” was translated into “practice”, in the case of BACE1 inhibitors this was not applicable. BACE1 inhibitors effectively reduce Aβ synthesis; however, this doesn’t affect either the clinical progression nor alleviate cognitive symptoms in AD [5,7]. These unexpected results might have some explanations, as summarized below.

First, the knowledge of BACE1 biology and catalytic mechanisms is incomplete; thus, it is possible that more efficient inhibitors can be provided in the future when some unknown aspects of BACE1 biology will be revealed.

Second, BACE1 inhibitors may be useful only in patients with overt increase in enzyme activity. In this context, the measurement of blood BACE1 could be a first-step screening tool for the selection of patients (with high plasma BACE1 and Aβ) to be included into clinical trials.

Third, the accumulation of Aβ in the brain doesn’t depend only on BACE1 activity, but rather on the balance between synthesis and clearance of Aβ [5]. Thus, reducing BACE1 activity may not actually reduce Aβ deposition if its clearance is also compromised.

Fourth, the amyloidogenic process cannot represent the only target for the treatment of a complex disease such as AD. This growing awareness has prompted the investigation of combination therapies (pharmacological trials) targeting, besides Aβ, other AD core pathological mechanisms like neuroinflammation and tau [8].

Fifth, an increase in BACE1 activity might be only a risk factor for the development of AD; of consequence, the association between high Aβ production and AD would be much more complex and not simply causative.

Finally, to be effective a treatment should be provided to patients when it can give a real health benefit. For example, it is well known that the efficacy of statins in reducing cardiovascular events in elderly patients is much reduced compared to young/adult ones. Similarly, the inhibition of BACE1 would be much less effective in delaying/slowing down AD in the elderly, as a consequence of a more advanced stage of pre-clinical/clinical AD, ageing brain, lower impact of BACE1 in AD pathogenesis in the elderly or longer time for possible regression of the disease.

It is worth it to mention that a pathological biomarker cannot always be a therapeutic target; therefore, although BACE1 has the credentials to be a good marker for AD, the efficacy of its inhibition in improving AD clinical features has not been proven.

Instead, since BACE1 plays a key role in Aβ42 production, it is possible that its increased activity may become an early-stage or high-risk marker for the pre-clinical stage of AD.

## References

[1] O’Brien RJ, Wong PC. Annu Rev Neurosci. 2011; 34:185–204. 10.1146/annurev-neuro-061010-113613 21456963 PMC3174086

[2] Cervellati C, et al. Geroscience. 2020; 42:159–67. 10.1007/s11357-019-00127-6 31745860 PMC7031490

[3] Zuliani G, et al. Sci Rep. 2020; 10:14980. 10.1038/s41598-020-72168-3 32917964 PMC7486910

[4] Vergallo A, et al.> Alzheimers Dement. 2021; 17:629–40. 10.1002/alz.12228 33527718

[5] Hampel H, et al. Biol Psychiatry. 2021; 89:745–56. 10.1016/j.biopsych.2020.02.001 32223911 PMC7533042

[6] Ashton NJ, et al. Eur J Nucl Med Mol Imaging. 2021. 10.1007/s00259-021-05253-y 33677733 PMC8175325

[7] Hrabinova M, et al. Curr Neuropharmacol. 2021; 19:61–77. 10.2174/1570159X18666200503023323 32359337 PMC7903497

[8] Cummings J, et al. Alzheimers Dement (N Y). 2019; 5:272–93. 10.1016/j.trci.2019.05.008 31334330 PMC6617248

